# Ectopic Expression of the Coleus R2R3 MYB-Type Proanthocyanidin Regulator Gene *SsMYB3* Alters the Flower Color in Transgenic Tobacco

**DOI:** 10.1371/journal.pone.0139392

**Published:** 2015-10-08

**Authors:** Qinlong Zhu, Shunzhao Sui, Xinghua Lei, Zhongfang Yang, Kun Lu, Guangde Liu, Yao-Guang Liu, Mingyang Li

**Affiliations:** 1 State Key Laboratory for Conservation and Utilization of Subtropical Agro-bioresources, College of Life Sciences, South China Agricultural University, Guangzhou 510642, China; 2 Chongqing Engineering Research Center for Floriculture, College of Horticulture and Landscape, Southwest University, Chongqing 400716, China; 3 Department of Botany, Chongqing Agricultural School, Chongqing 401329, China; 4 Chongqing Engineering Research Center for Rapeseed, College of Agronomy and Biotechnology, Southwest University, Chongqing 400716, China; 5 ChongqingAgricultural Broadcasting and Television School, Chongqing 401121, China; Wuhan Botanical Garden of Chinese Academy of Sciences, CHINA

## Abstract

Proanthocyanidins (PAs) play an important role in plant disease defense and have beneficial effects on human health. We isolated and characterized a novel R2R3 MYB-type PA-regulator *SsMYB3* from a well-known ornamental plant, coleus (*Solenostemon scutellarioides*), to study the molecular regulation of PAs and to engineer PAs biosynthesis. The expression level of *SsMYB3* was correlated with condensed tannins contents in various coleus tissues and was induced by wounding and light. A complementation test in the *Arabidopsis tt2* mutant showed that *SsMYB3* could restore the PA-deficient seed coat phenotype and activated expression of the PA-specific gene *ANR* and two related genes, *DFR* and *ANS*. In yeast two-hybrid assays, SsMYB3 interacted with the *Arabidopsis* AtTT8 and AtTTG1 to reform the ternary transcriptional complex, and also interacted with two tobacco bHLH proteins (NtAn1a and NtJAF13-1) and a WD40 protein, NtAn11-1. Ectopic overexpression of *SsMYB3* in transgenic tobacco led to almost-white flowers by greatly reducing anthocyanin levels and enhancing accumulation of condensed tannins. This overexpression of *SsMYB3* upregulated the key PA genes (*NtLAR* and *NtANR*) and late anthocyanin structural genes (*NtDFR* and *NtANS*), but downregulated the expression of the final anthocyanin gene *NtUFGT*. The formative SsMYB3-complex represses anthocyanin accumulation by directly suppressing the expression of the final anthocyanin structural gene *NtUFGT*, through competitive inhibition or destabilization of the endogenous NtAn2-complex formation. These results suggested that SsMYB3 may form a transcription activation complex to regulate PA biosynthesis in the *Arabidopsis tt2* mutant and transgenic tobacco. Our findings suggest that *SsMYB3* is involved in the regulation of PA biosynthesis in coleus and has the potential as a molecular tool for manipulating biosynthesis of PAs in fruits and other crops using metabolic engineering.

## Introduction

**Fig 1:**
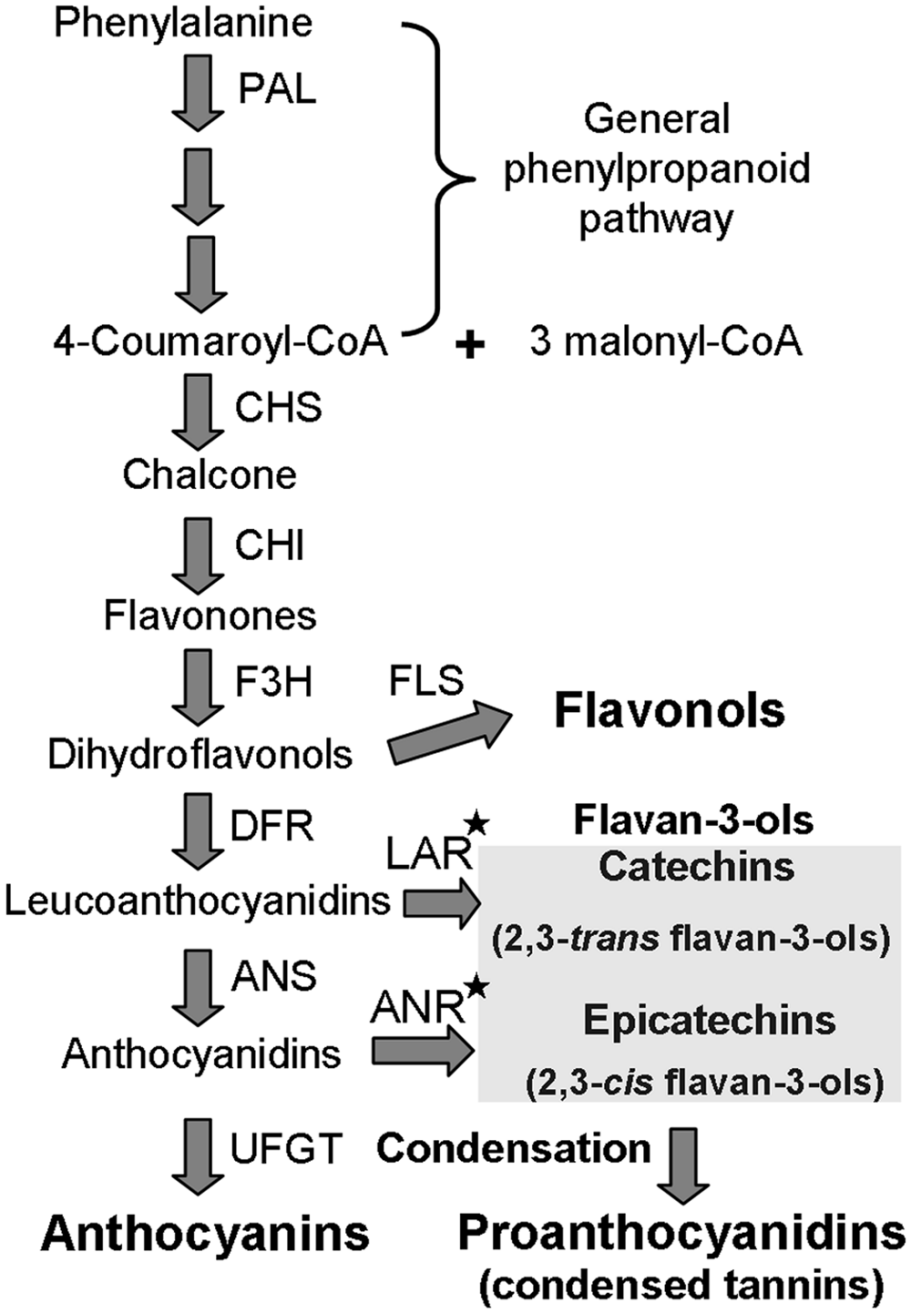
Schematic diagram of the flavonoid biosynthesis pathway, including main branches of anthocyanins, PAs, and flavonols. PAL, phenylalanine ammonia lyase; CHS, chalcone synthase; CHI, chalcone isomerase; F3H, flavonone 3-hydroxylase; DFR, dihydroflavonol reductase; FLS, flavonol synthase; ANS, anthocyanin synthase; LAR, leucoanthocyanidin reductase; ANR, anthocyanidin reductase; UFGT, UDP-glucose: flavonoid 3-*O*-glucosyltransferase. Asterisks indicate key structural enzymes in PA biosynthesis.

Proanthocyanidins (PAs), also called condensed tannins (CTs), result from condensation of flavan-3-ols, and belong to one of the main classes of polyphenolic compounds synthesized via the flavonoid biosynthetic pathway [1]. PAs are widely present in the plant kingdom, in fruits, seeds, flowers, leaves, and bark, and play an important role in defense against plant diseases and herbivores [2]. PAs contribute to the quality and health benefits of many important plant products, such as fruit and wine [3]. As powerful antioxidants, PAs can provide multiple beneficial effects for human health, including enhanced immunity and protection against free radical-mediated injury and cardiovascular diseases [4, 5]. For these reasons, it is important to further understand the molecular regulation and metabolic engineering of PAs to improve the nutrient and health values of important crops and fruits. As a branch of the flavonoid pathway, PA biosynthesis shares almost all structural genes with anthocyanin biosynthesis, except for the last catalytic steps (Fig 1).

Synthesis of PAs begins with the generation of flavan-3-ol units (e.g., catechin and epicatechin); two key structural genes and one R2R3 MYB-type regulatory gene involved in PAs biosynthesis have been identified. The key enzymes are leucoanthocyanidin reductase (LAR, EC 1.17.1.3) and anthocyanidin reductase (ANR, EC 1.3.1.77), which convert leucocyanidin and cyanidin to catechin and epicatechin, respectively. The molecular and biochemical functions of the genes encoding these enzymes have been identified in several plant species, for example, *LAR*s in apple [5], legume[6], grapevine (*Vitis vinifera*)[3, 7], and *Medicago truncatula* [8]; and *ANR*s in *Arabidopsis*[9], grapevine [3, 7], tea [10], apple [11], and soybean [12]. In flavonoid biosynthesis, the transcriptional activities of the key enzymes are controlled by R2R3 MYB transcription factors (TFs) combined with bHLH and WD40 proteins that form a MYB-bHLH-WD40 (MBW) transcriptional activation complex in which the MYBs play a crucial role [1, 13–15]. To date, functional characterization of the MYBs involved in the production of PAs has been reported in *Arabidopsis* (*AtTT2*) [16], grapevine (*VvMYBPA1* and *VvMYBPA2*) [17, 18], *Lotus japonicus* (*LjTT2a/b/c*) [19], persimmon (*DkMYB2* and *DkMYB4*) [20, 21], poplar (*PtMYB134*) [22], and *Medicago truncatula* (*MtPAR*) [23]. For example, in *Arabidopsis*, the MBW transcription complex of PA regulation is composed of three *TRANSPARENT TESTA* (*TT*) genes: *AtTT2* (AtMYB123), *AtTT8* (AtbHLH042), and *AtTTG1* (WD40-repeat protein) [16, 24]. The *AtANR* transcripts could not be detected in the *tt2* mutant, and PAs cannot accumulate in the seed coat. In grapevines, *VvMYBPA1* and *VvMYBPA2* regulate PA synthesis during fruit development and significantly activate *VvLAR1*, *VvANR*, and several flavonoid structural genes [17, 18]. In persimmon fruits, *DkMYB4* regulates the expression of *DkANR* and has little influence on *DkLAR*, but *DkMYB2* can directly activate both of these key genes, which are induced by wound stress [20, 21].

Ectopic overexpression of the key enzyme-encoding genes of the PA pathway has been utilized to engineer CT biosynthesis in tobacco. However, there are few reports on the use of the MYB-type PA-regulation method. Although ectopic expression of *ANR*s from *Arabidopsis* [9], grapevine [7], and *Medicago truncatula* [11], led to loss of anthocyanins and accumulation of CTs, overexpression of *MtLAR* did not increase the content of CTs in transgenic tobacco flowers [8]. These results indicate the limitations and uncertainty of the single key enzyme strategy. In contrast, MYBs are more suitable for engineering CT biosynthesis because of their regulation of multiple structural genes. Therefore, isolation and utilization of novel MYBs involved in the PA pathway will provide valuable insights into the molecular regulation and metabolic engineering of PA biosynthesis in plants.

Coleus, *Solenostemon scutellarioides* (L.) Codd, is a well-known ornamental plant with colorful foliage and is a popular houseplant worldwide [24, 25]. Because it is rich in secondary metabolites (e.g., rosmarinic acid and flavonoids), coleus is used as a medical plant in countries such as India, Indonesia, and Mexico [26]. Rosmarinic acid is an ester of caffeic acid involved in plant defense and antioxidant activities. The biosynthesis pathway and related genes for rosmarinic acid have been characterized in coleus [27, 28]. However, few studies have reported on the key structural and regulatory genes involved in the flavonoid pathway (especially in biosynthesis of PAs and anthocyanins) in coleus. Therefore, isolation and characterization of these regulatory genes, such as MYBs, will be necessary for understanding the molecular regulation of biosynthesis of PAs or anthocyanins in this species.

In this study, we isolated and characterized a regulatory gene in coleus, *SsMYB3*, which encodes an R2R3 MYB TF. The expression level of *SsMYB3* is correlated with CT content in various tissues of coleus and is induced by wounding and light. A complementation test of this TF gene in the *Arabidopsis tt2* mutant shows that *SsMYB3* could restore the PA-deficient seed coat phenotype. Ectopic overexpression of *SsMYB3* leads to a large reduction in anthocyanin level and enhanced accumulation of CTs in transgenic tobacco flowers. These results suggest that *SsMYB3* is a functional PA regulator and may be a useful molecular tool for metabolic engineering of PA biosynthesis in plants.

## Materials and Methods

### Plant materials, stress treatments, and isolation of total RNA

The red-leaved coleus variety ‘Red Trailing Queen’ was grown in a greenhouse under natural light conditions at 25°C. Wild-type and T1 transgenic tobacco (*Nicotiana tabacum* W38) plants were grown under the same conditions. The *Arabidopsis tt2* mutant was purchased from ABRC (Arabidopsis Biological Resource Center, OH, USA) and was used for complementation analysis.

Three stress treatments were performed for gene-expression studies. In a dark experiment, coleus seedlings were cultured in the dark and leaves were collected at 0, 1, 8, 24, and 48 h after treatment. After 2 weeks continuous culture under dim light (200 lux), leaves of the coleus seedlings were exposed to high light intensity (25,000 lux) and collected at 0, 0.5, 1, 2, and 8 h after lighting. For wounding treatments with reference to [22], the edges of coleus leaves were crushed with pliers. All the required samples were immediately frozen in liquid nitrogen and stored at –80°C until analysis.

Total RNA was isolated from frozen samples using a W6711 Total RNA Extraction Kit (Watson, China) or Trizol reagent (Invitrogen, USA) according to the manufacturer’s instructions, followed by incubation with RNase-free DNase I (Takara, Dalian, China).

### Cloning of the full-length *SsMYB3* gene

First-strand cDNA was synthesized from 1 μg of DNase-treated RNA with an M-MLV reverse transcriptase kit (Promega, USA) in a total volume of 20 μL, using oligo (dT) 15 primer. The numerous fragments encoding parts of the R2R3 MYB domains were obtained from young red coleus leaves using degenerate oligonucleotide primer PCR. The degenerate primers, FdMYB and RdMYB, were designed according to the conserved regions of the coleus R2R3 MYBs involved in flavonoid biosynthesis.

One cDNA clone, *SsMYB3*, showed high similarity to other known flavonoid regulators and was selected to isolate the full-length cDNA sequence using RACE PCR (GeneRacer Kit, Invitrogen, USA). The full-length cDNA and DNA was then amplified with two specific primers, FMYB3 and RMYB3, in a total volume of 50 μL with the following protocol: 94°C for 4 min; 35 cycles of 94°C for 30 s; 56°C for 30 s; and 72°C for 2 min; followed by a final extension of 72°C for 5 min. All amplified products were purified, subcloned, and sequenced. The primers used for the degenerate PCR, RACE PCR, and the full-length amplification of the *SsMYB3* gene are listed in S1 Table.

### Sequence analysis and construction of expression vector

Molecular characterizations and multiple sequence alignments of *SsMYB3* were analyzed using the Vector NTI 10.0 software package (Invitrogen, USA). For phylogenetic analysis, several full-length amino acid sequences of R2R3 MYBs involved in regulation of flavonoid metabolism were selected for alignment using the ClustalX 1.8 program with default parameters, and the tree was constructed using the neighbor-joining method in the MEGA 5.0 package [29].

The modified plant binary vector pCAMBIA2301G was constructed by inserting an expression cassette with CaMV 35S promoter, multiple cloning sites (*Xba*I, *Bam*HI, *Sma*I, *Kpn*I, and *Sac*I) and NOS terminator into the *Eco*RI and *Hin*dIII sites of pCAMBIA2301; the PCR product of *SsMYB3* cDNA with *Xba*I and *Sma*I sites was then subcloned into the same sites of pCAMBIA2301G. The resulting vector, pCAMBIA2301G-SsMYB3, containing a plant-selectable marker *NPTII* gene conferring kanamycin resistance, was sequenced and then transferred into *Agrobacterium tumefaciens* strain LBA4404 by the freeze-thaw method [30].

### *Arabidopsis* complementation analysis

The *Arabidopsis tt2* mutant lacking PA biosynthesis in the seed coat was transformed with *A*. *tumefaciens* strain LBA4404 containing the binary construct pCAMBIA2301G-SsMYB3 by floral dipping [31]. Harvested seeds were selected on MS medium containing 3% (v/v) sucrose and with 100 mg/L kanamycin. For phenotypic analysis of CT accumulation in the seed coat, T_1_ seeds were stained with dimethylaminocinnaldehyde reagent (DMACA, Sigma) according to a previous report [32].

### Transformation of tobacco plants

The *Agrobacterium* strain LBA4404 containing the binary vector was incubated in liquid YEB medium supplemented with 200 mmol/L acetone-syringone at 28°C. The *Agrobacterium* was co-cultivated with leaf discs from *N*. *tabacum* cv.W38 until OD_600_ reached 0.5–0.6, and the transformation method according to a previous report [33]. Transformed plants were grown on MS medium containing 100 mg/L kanamycin under a 16-h photoperiod at 25°C. Finally, transgenic tobacco plants were identified by PCR amplification of the *NPTII* and *SsMYB3* genes of kanamycin-resistant plantlets.

### Yeast two-hybrid analysis

To detect proteins that may interact with SsMYB3 in *Arabidopsis* and tobacco, yeast two-hybrid (Y2H) assays were performed, using the Matchmaker™ Gold Y2H System (Clontech, USA). For the Y2H experiments, the full-length coding sequence of *SsMYB3* was ligated into the pGADT7 (activation domain, AD) and pGBKT7 (binding domain, BD) vectors. The full-length cDNAs of *AtTT2*, *AtTTG1*, and *NtAn11-1* (WD40), and the coding sequences of MYB-interaction regions of *AtTT8* (aa1-204), *NtAn1a* (aa1-195), or *NtJAF13-1* (aa1-203) were ligated into the pGBKT7 (BD) vector using previous method [34]. The AD and BD fusion vectors were co-transformed into the *Saccharomyces cerevisiae* strain Y2HGold using the lithium-acetate method as described in the Clontech yeast protocol handbook. Co-transformed colonies were first selected on SD medium lacking leucine and tryptophan (SD-Leu–Trp), and were then screened for growth on quadruple-selection SD medium lacking adenine, histidine, leucine, and tryptophan (SD-Ade–His–Leu–Trp).

### Expression analysis of RT-PCR and quantitative real-time PCR (qPCR)

Isolation of total RNA from different plant materials and synthesis of first-strand cDNA was performed as described above.

Expression of the *SsMYB3* gene and several key flavonoid genes in *SsMYB3*-overexpressing *Arabidopsis* immature siliques and tobacco flowers were determined by RT-PCR using the following program: 94°C (4 min); 18–22 cycles of 94°C (30 s), 58°C (30 s), and 72°C (30 s); and 72°C (5 min). All primers used for RT-PCR are listed in S2 Table. The transcript levels of the *SsMYB3* gene in different coleus tissues and in leaves subjected to the different treatments were measured by qPCR. The coleus actin gene *SsACT* was used as an internal control to normalize gene expression. The results were calculated as differences in cycle threshold (Ct) between *SsMYB3* and actin genes (2^-ΔCt^). The expression of the structural genes of anthocyanin and PA pathways was measured by qPCR using RNA isolated from the *SsMYB3*-overexpressing tobacco flowers. The tobacco *GAPDH* gene was used as an endogenous control to normalize gene expression. The results were analyzed using the comparative Ct method and quantified relative to the wild type (2^-ΔΔCt^).

All qPCR assays were performed using SYBR Green Master Mix reagent (TaKaRa, Dalian, China) using a BioRad IQ5 real-time PCR detection system, following thermal cycling conditions recommended by the manufacturer. All PCR reactions were performed in triplicate and repeated twice. The primer sequences for qPCR are listed in S3 Table.

### Measurement of anthocyanin and condensed tannins

Quantification of anthocyanin was performed as described by Rabino *et al*. [35, 36] with some modifications. Briefly, fresh flowers of *SsMYB3*-overexpressing tobacco were ground in liquid nitrogen and placed into 5 mL extraction buffer (1% HCl in methanol) overnight in the dark at 4°C. After centrifugation, 1 mL of extract was diluted with1 mL of water, 2 mL of chloroform was added to remove chlorophyll, and the mixture was centrifuged for 10 min at 12,000 × g. Absorption of the aqueous phase was measured spectrophotometrically at 530 and 650 nm. The anthocyanin content was quantified as (A530–0.25 × A650) g^-1^ fresh weight (FW). The experiment was repeated three times for each transgenic plant.

The amount of soluble CTs from leaves of coleus or wild-type and transgenic tobacco flowers was determined by the vanillin-HCl method as reported previously [37, 38]. Leaves or flowers were ground in liquid nitrogen, extracted with 10 mL of methanol, and shaken for 20 min following centrifugation. One milliliter of supernatant was incubated with 5 mL vanillin-HCl reagent (0.5% vanillin solution in methanol containing 4% HCl [v/v]) for 30 min at 30°C. The reaction solution was determined at 500 nm using a UV-VIS spectrophotometer (Shimadzu, Japan). The mean CT content was obtained from three replicate measurements of each plant material.

## Results and Discussion

### Isolation and characterization of *SsMYB3* gene

On the basis of the conserved sequences of R2R3 MYBs from *ZmP* (U57002), *AtMYB12* (AEC10843), *AtPAP1* (ABB03879), *PhAN2* (AF146702), and *GmYB10* (AJ554700), we designed degenerate PCR primers to amplify an approximately 200-bp fragment using cDNA from coleus leaves as a template. Following subcloning and sequencing of the PCR product, nine different DNA fragments were obtained from 20 independent clones. One of which, named *SsMYB3*, had similarity with the conserved region of MYBs related to flavonoid synthesis. According to this partial sequence, the complete cDNA sequence of the *SsMYB3* gene was determined by 5′- and 3′-RACE PCR. Then, the genomic sequence of *SsMYB3* was amplified and sequenced (S1 Fig).The GenBank accession numbers of the sequences are EF522163 and EF522164, respectively.

**Fig 2:**
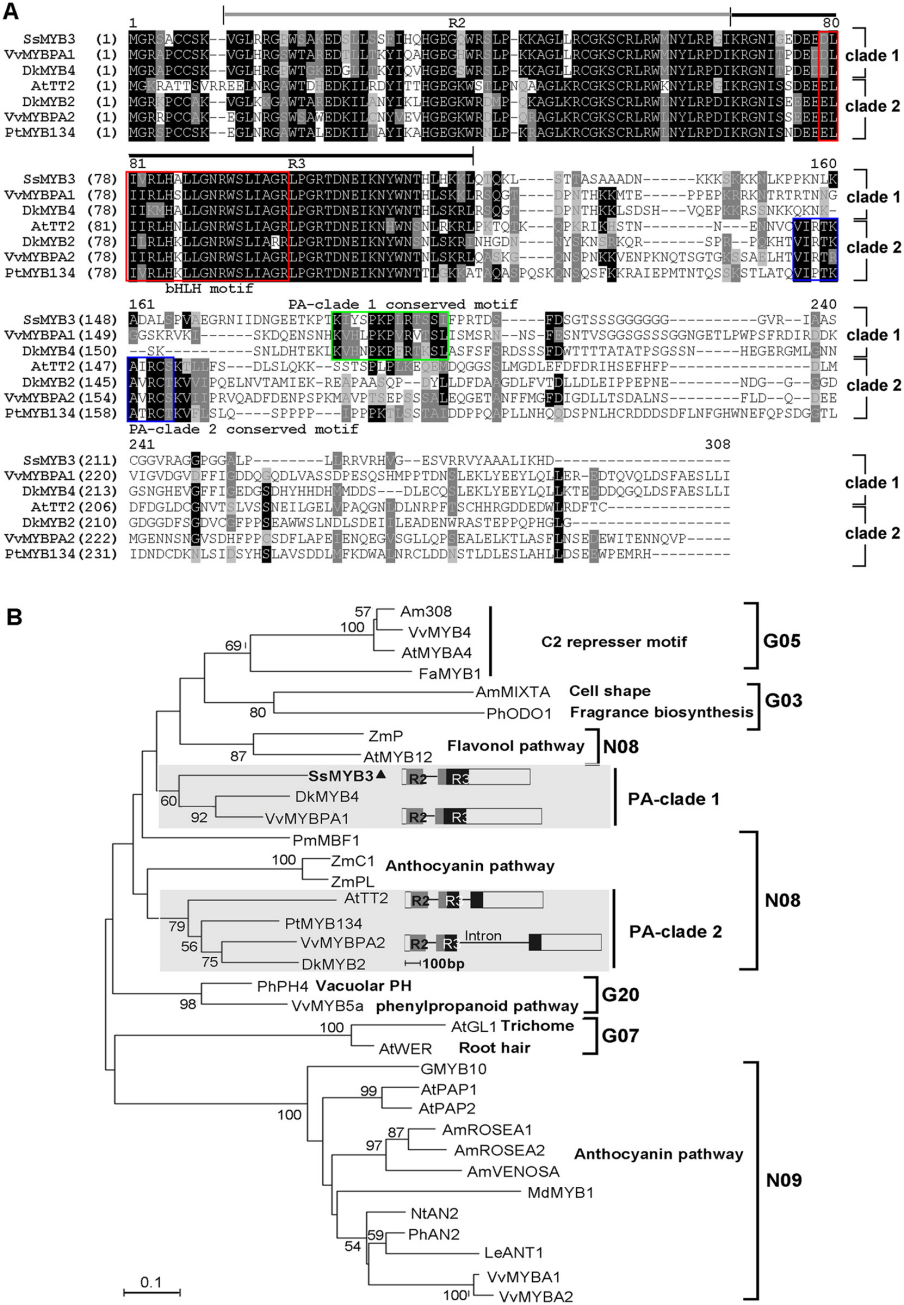
Comparison of SsMYB3 with other functional R2R3-MYB TFs. **(A)** Full-length protein sequence alignments of the MYBs by Vector NTI 10.0 software. The R2 and R3 domains are shown; the bHLH motif [D/E]Lx_2_[R/K]x_3_Lx_6_Lx_3_R and two possible PA specifically conserved motifs, K[I/V]x_2_PKPx_1_Rx_2_S[I/L] and VI[R/P]TKAx_1_RC[S/T], are indicated in the green and blue box, respectively. **(B)** Phylogenetic tree analysis depicts SsMYB3 belonging to PA-clade 1. The tree was constructed from the ClustalX 1.8 alignment using the neighbor-joining method in the MEGA 5.0 program. Scale bar represents 0.1 substitutions per site, and numbers next to nodes are bootstrap values from 1,000 replicates (only values >50% are shown). SsMYB3 and other known functional PA-biosynthesis regulators are divided into two subclades (PA-clades 1 and 2) with different genomic structures indicated in gray background. MYB proteins with known functions and MYB subgroups (G03, G05, G07, G20, N08, and N09) are indicated. GenBank accession numbers of MYBs in the phylogenetic tree are as follows: AmMIXTA (CAA55725), AmROSEA1 (ABB83826), AmROSEA2 (ABB83827), AmVENOSA (ABB83828), Am308 (JQ0960), AtTT2 (Q9FJA2), AtPAP1 (ABB03879), AtPAP2 (NP_176813), AtMYB4 (BAA21619), AtMYB12 (ABB03913), AtWER (AAF18939), AtGL1 (AAC97387), DkMYB2 (AB503699), DkMYB4 (AB503671), FaMYB1 (AAK84064), GMYB10 (CAD87010), LeANT1 (AAQ55181), MdMYB1 (DQ886414), PmMBF1 (AAA82943), PhAN2 (AAF66727), PhPH4 (AAY51377.1), PhODO1 (AAV98200), PtMYB134 (ACR83705), ZmC1 (AAA33482), ZmPl (AAA19821), ZmP (AAC49394), VvMybPA1 (CAJ90831), VvMybPA2 (EU919682), VvMYBPA1 (BAD18977), VvMYBPA2 (BAD18978), VvMYB4 (ABL61515), and VvMYB5a (AAS68190).

The full-length cDNA sequence was 826 bp in size, containing a 747-bp ORF; the corresponding genomic sequence was 931 bp and consisted of two exons and a 105-bp intron with a standard GT/AG splicing site (S2 Fig). *SsMYB3* encoded a 248 amino acid protein with predicted molecular mass of 27 kDa and calculated isoelectric point of 10.42. Using the NCBI conserved domain search program (http://www.ncbi.nlm.nih.gov/Structure/cdd/wrpsb.cgi), an N-terminal R2R3 repeat corresponding to the DNA-binding (MYB) domain was detected in the deduced amino acid sequence of SsMYB3 (Fig 2A). Similar to related MYBs, the bHLH motif [D/E]Lx_2_[R/K]x_3_Lx_6_Lx_3_R that interacts with bHLH proteins was identified in the highly conserved N-terminal R2R3 region of SsMYB3 (Fig 2A). Despite the fact that C-terminal regions of MYBs are highly variable, a novel, possibly conserved motif, K[I/V]x_2_PKPx_1_Rx_2_S[I/L], was found only in SsMYB3 and in two other members of PA-clade 1 (Fig 2A) by alignment with MYBs of known function that regulate PA biosynthesis, using Vector NTI 10.0 software. At the same time, the C-terminal conserved motif in AtTT2, VI[R/P]TKAx_1_RC[S/T] [16], was found only in members of PA-clade 2. The MYB domain and full-length sequence of SsMYB3 were most closely related to the grapevine PA-synthesis regulator VvMYBPA1, with 83% and 60% identical amino acid residues, respectively.

To further investigate the homology of SsMYB3 to 33 function-known plant-R2R3 MYB-type proteins, a phylogenetic tree was constructed using the full-length amino acid sequences. These MYBs were clustered into distinct groups according to their functions and structural characterizations (Fig 2B). All MYBs characterized as PA regulators could be placed in two separate subclades: PA-clade 1 contained SsMYB3, VvMYBPA1, and DkMyb4; PA-clade 2 contained AtTT2, PtMYB134, VvMYBPA2, and DkMyb2. The result of this phylogenetic analysis was consistent with previous reports on PA regulators PtMYB134 [22] and DkMyb2 [21]. In addition, by comparative analysis of the genomic structure of several PA-regulatory genes, we found that the two PA clades had different gene structures: genes in PA-clade 1 had one intron, while those in PA-clade 2 had two (Fig 2B). Previous studies have shown that the two types of PA regulators have different expression patterns. For examples, in *V*. *vinifera*, *VvMYBPA1* [17] and *VvMYBPA2* [18], which are from PA-clade 1 and 2, respectively, have different expression profiles in seeds, skin and leaves. In persimmon (*Diospyroskaki*), *DkMyb4* [20] and *DkMyb2* [21] belong to PA-clade 1 and 2, and are orthologs of *VvMYBPA1* and *VvMYBPA2*, respectively. The expression level of *DkMyb4* is considerably higher than that of *DkMyb2* in each plant organ. Furthermore, the PA-clade 1 regulators, such as VvMYBPA1 and DkMyb4, mainly recognized the MYBCORE *cis*-motif, but PA-clade 2 regulators, such as DkMyb2 and PtMYB134, mainly recognized the AC element of MYB-binding *cis*-motif, in the promoter regions of PA pathway genes.

These differences in gene structure, protein C-terminal conserved motifs, expression patterns and identification of conserved motifs implied that the two PA regulatory clades controlled the transcription of PA-specific genes in different ways, which might form diverse regulatory strategies in plant PA biosynthesis. These results strongly suggested that *SsMYB3* is a PA-clade 1 regulatory gene.

### Expression of *SsMYB3* is correlated with PA accumulation and is induced by light and wounding in coleus

**Fig 3:**
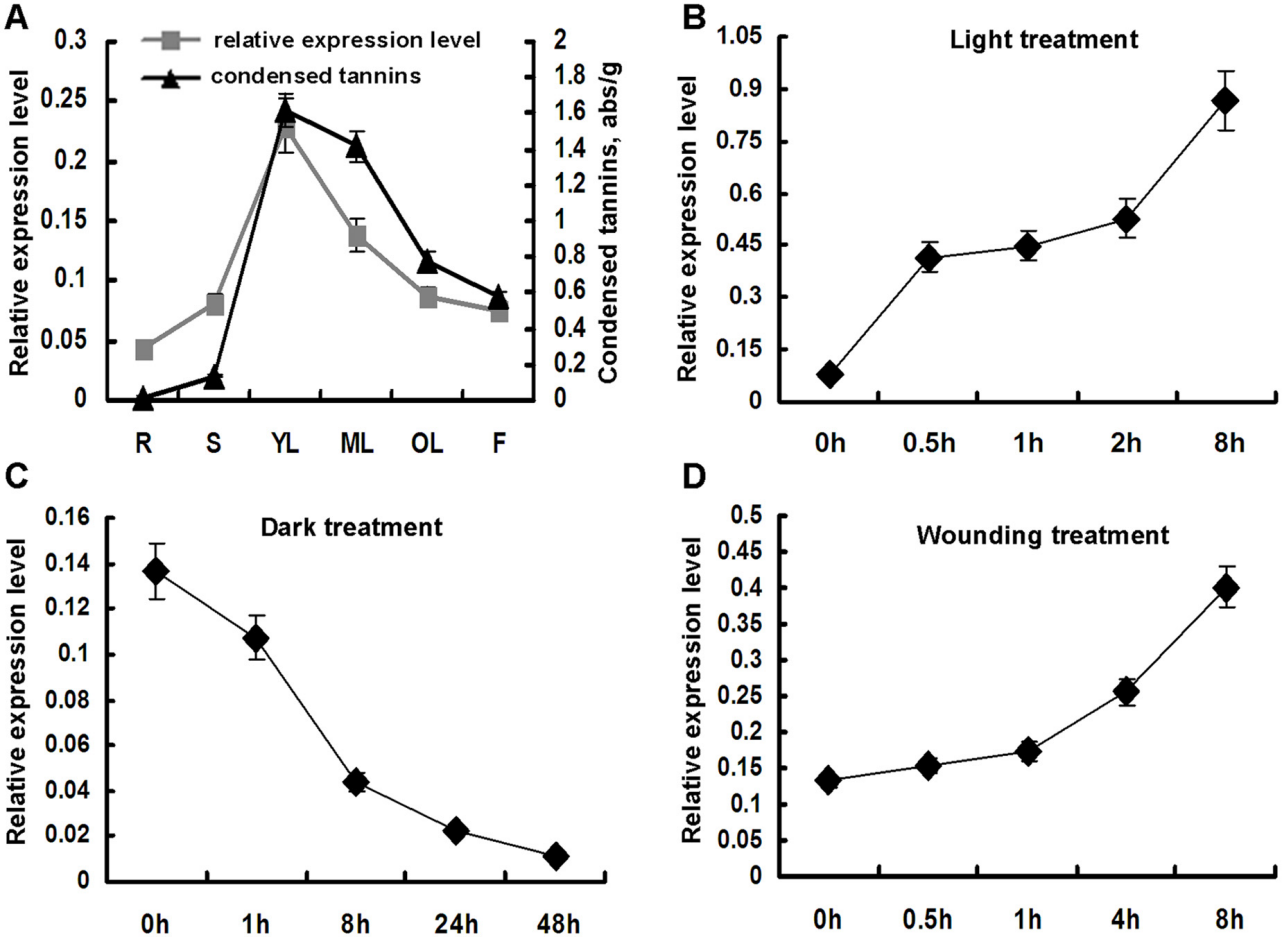
Accumulation of PAs and the gene expression profiles of *SsMYB3*. **(A)** The expression pattern of *SsMYB3* and accumulation of PAs in various tissues of coleus: R (root), S (stem), YL (young leaf), ML (mature leaf), OL (old leaf), and F (flower). Transcription pattern of *SsMYB3* in leaves under different treatments: **(B)** light, **(C)** dark, and **(D)** wounding. All expression data were normalized to the coleus *SsActin* gene, and values represent averages of three technical replicates.

To confirm the relationship between expression of *SsMYB3* and PA accumulation, qPCR expression analysis of the gene and determination of PA content were performed in different tissues of coleus. *SsMYB3* was expressed in all tested tissues, with highest level in young leaves (YL) and lowest level in roots (R) (Fig 3A). The *SsMYB3*expression declined gradually in leaves as developed and matured. The concentration of PAs changed in accordance with the changing level of gene expression in different coleus tissues. These results indicated that the expression pattern of *SsMYB3* was consistent with the accumulation of PAs in coleus tissues. Similar results were described for PA regulators in grapevine (*VvMYBPA1*) [17] and persimmon (*DkMyb4*) [20].

Current studies have shown that visible light primarily induces biosynthesis of PAs in grapevine by upregulating expression of the key genes involved in this pathway [39]. To investigate whether light induces *SsMYB3* expression, dark- and light-induction treatments were performed. When coleus leaves were exposed to high intensity of light, transcript numbers of *SsMYB3* increased rapidly to approximately 5-fold higher at 0.5 h than at 0 h (CK) and continued to increase at 8 h treatment (Fig 3B). In the dark treatment, the expression level of *SsMYB3* decreased gradually during the first hour and reached a minimum at 48 h dark (Fig 3C). These results demonstrated that *SsMYB3* expression is light-inducible, and suggested that light may be an important factor for PA biosynthesis in coleus. Because several PA regulators (e.g., *PtMYB134*, *DkMyb2*, and *DkMyb4*) are reported to be induced by wounding [18, 21], the possibility of wound induction of *SsMYB3* was explored; the transcript level of *SsMYB3* clearly increased in the wound-stress treatment (Fig 3D). From this result, we speculated that other MYB-type PA regulators may be induced by wound stress, although the stress-induction mechanism is unclear and requires further investigation.

### Complementation of the *Arabidopsis tt2* mutant

In the *Arabidopsis tt2* mutant, the MBW ternary transcription complex (AtTT2-AtTT8-AtTTG1) could not be formed and PA-specific *AtANR* gene could not be activated because of the loss of function of *AtTT2* [16, 24]. The lack of PAs in the *tt2* mutant seed coat creates the yellow-colored seed phenotype.

**Fig 4:**
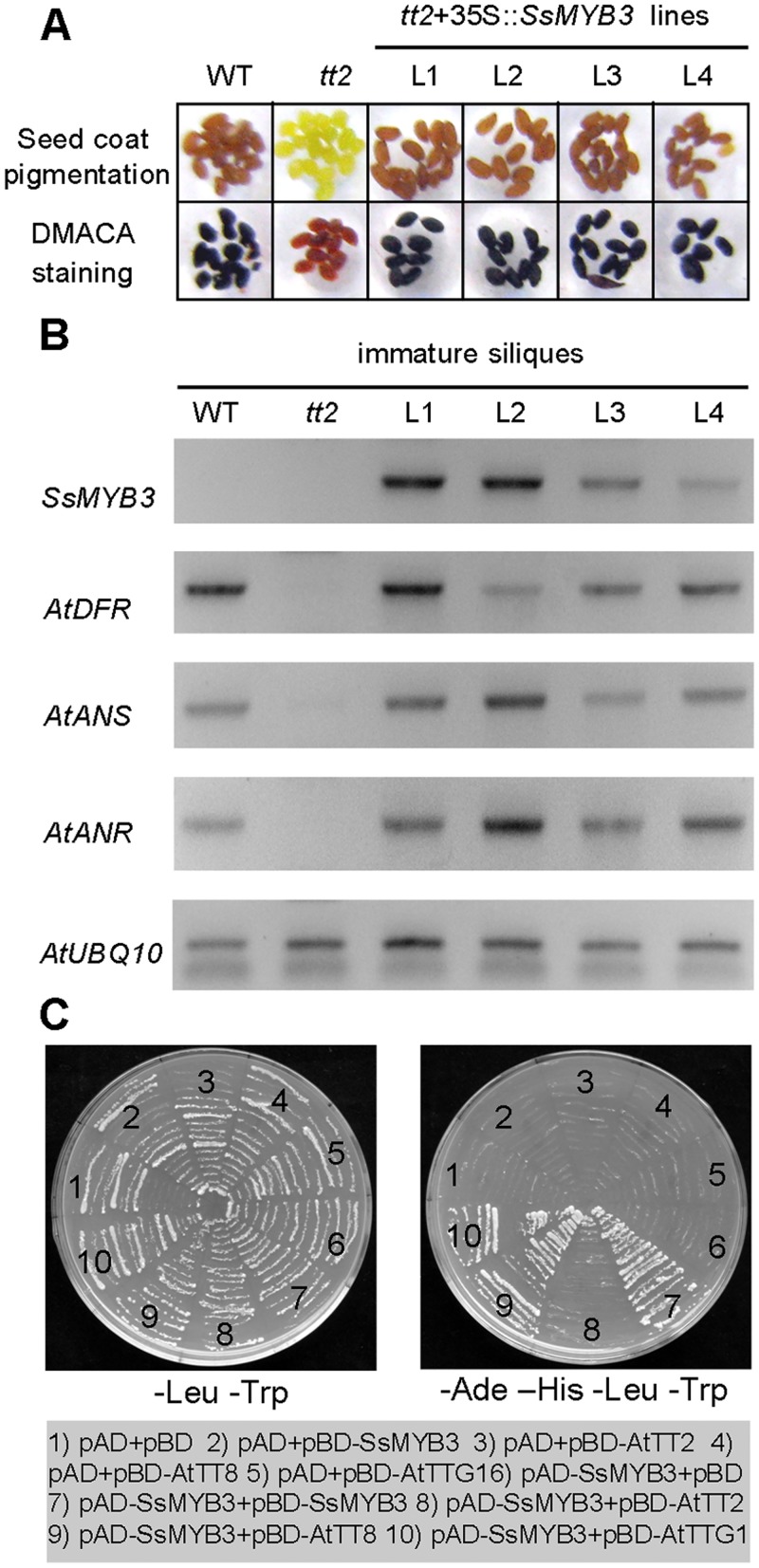
Complementation of *Arabidopsis* PA-deficient *tt2* mutant seed phenotype by overexpression of *SsMYB3*. **(A)** Seed coat pigmentation and DMACA staining of wild-type (WT), *tt2* mutant, and *tt2* 35S::SsMYB3 seeds. Seeds of WT and *tt2* 35S::SsMYB3 show brown pigmentation due to the accumulation of PAs, and their seed coats display dark coloration from staining by DMACA. The PA-deficient *tt2* mutant seed coat is yellow and cannot be stained by DMACA. **(B)** RT-PCR analysis of key structural genes related to PA biosynthesis in immature siliques of WT, *tt2* mutant, and *tt2* 35S::SsMYB3 lines. **(C)** Protein–protein interactions between SsMYB3 and *Arabidopsis* PA-related regulator AtTT8 or AtTTG1. In a Y2H assay, SsMYB3 fused to the GAL4-activation domain (pAD-SsMYB3) and GAL4-DNA-binding domain (pBD-SsMYB3); pAD-SsMYB3 was co-transformed with fusion constructs of the GAL4 DNA-binding domain with the WD40 protein AtTTG1 (pBD-AtTTG1) and the MYB-interaction regions from AtTT8 (pBD-AtTT8^aa1-204^).

To test whether the cloned *SsMYB3* was a functional PA regulator, the *SsMYB3* gene, under the control of the CaMV35S promoter in pCAMBIA2301G, was introduced into the *tt2* mutant by *A*. *tumefaciens*-mediated transformation. The transgenic T_1_ seeds showed the wild-type brown-colored seed coat, which could be stained black using DMACA reagent to indicate the accumulation of CTs (Fig 4A), consistent with the complementary phenotype of the *tt2* seed coat using *AtTT2* [16] and *VvMYBPA1* [17]. DMACA is a useful and specific reagent for detecting PAs by its reaction with both PA monomers and polymers [40]. This result demonstrated that the *SsMYB3* overexpression can complement the *tt2* mutant seed coat phenotype. Although SsMYB3 and VvMYBPA1 are from PA-clade 1, they can complement the PA-clade 2 Arabidopsis *tt2* mutant, which indicated that the two types of PA-clade factors have the functional conservation to regulate PA biosynthesis.

Further RT-PCR analyses showed that the PA-specific gene *ANR* and two closely related anthocyanin structural genes *DFR* and *ANS* were induced in immature siliques of *tt2* 35S::*SsMYB3*T_1_ lines (Fig 4B), while these early structural genes (*AtCHS*, *AtCHI* and *AtF3H*) of flavonoid pathway have not been affected (S3 Fig). This was similar to *Arabidopsis* TT2 [16] and grapevine VvMYBPA1 [17], which control PA-specific genes and related *DFR* and *ANS* genes. In addition, Y2H assays revealed that SsMYB3 could interact with AtTT8 and AtTTG1 to reform the MBW complex and form a homodimer with itself (Fig 4C), similar to the role played by AtTT2 [24]. Taken together, our *Arabidopsis* complementation experiments provided direct evidence that *SsMYB3* is a functional MYB-type PA regulator.

### Functional characterization of the *SsMYB3* gene in tobacco

Recent studies have shown that TFs, with their potential ability to activate multiple structural genes, are more effective tools than single structural genes (encoding enzymes) for plant metabolic engineering [41]. For example, ectopic expression of *DkMyb4*, which increased or activated the expression of multiple structural genes involved in the PA or anthocyanin pathway, resulted in massive accumulation of PAs in the callus of kiwifruit (*Actinidia deliciosa*) [20]. However, similar approaches for regulating PA biosynthesis in the model plant tobacco have not yet been reported. And it is unknown that whether ectopic expression of MYB PA-regulators would affect the flower color.

**Fig 5:**
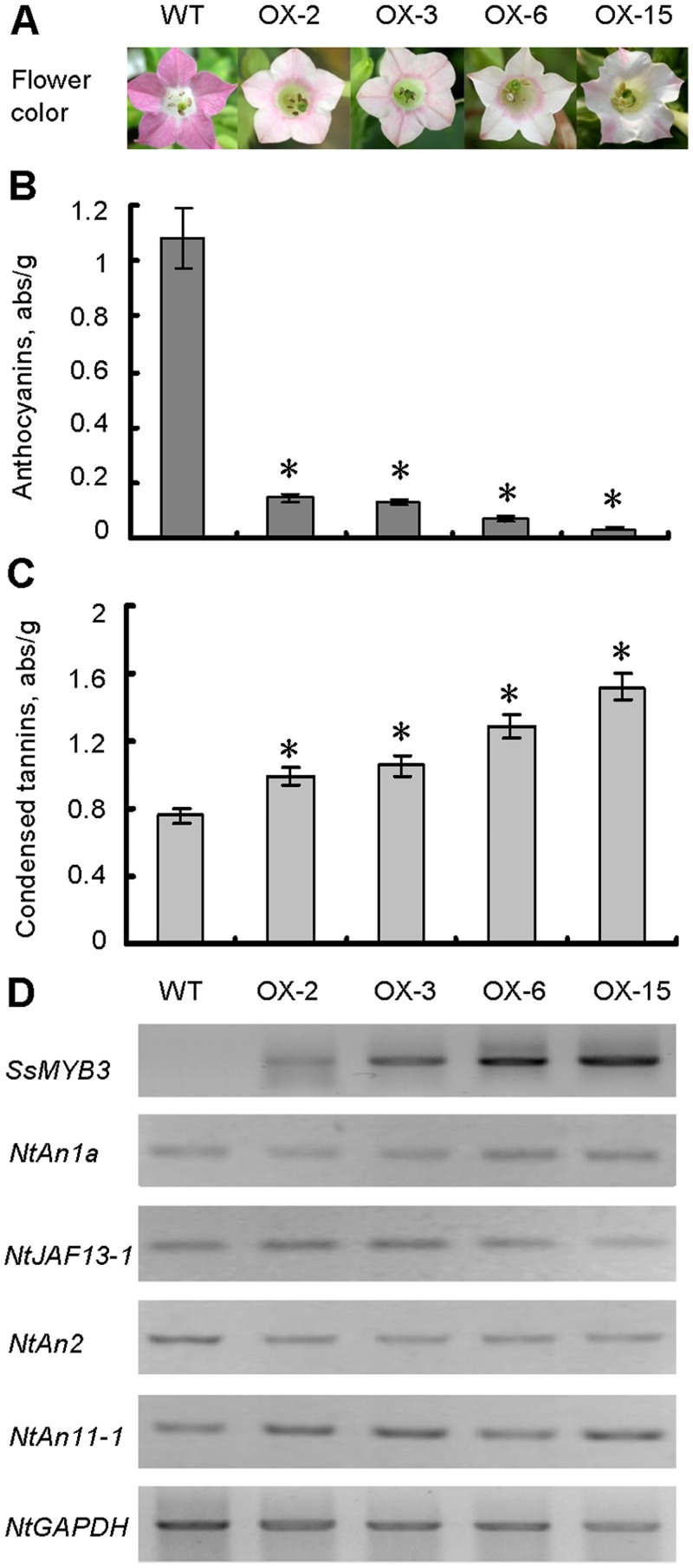
Functional characterization of *SsMYB3* by its ectopic expression in tobacco. **(A)** Overexpression of *SsMYB3* resulted in visibly decreased color in the corolla of transgenic tobacco flowers. **(B)** Relative anthocyanin contents quantified as (A530–0.25 × A650)/fresh weight (g). **(C)** The measured relative condensed tannin contents at 500-nm absorbance. **(D)** RT-PCR expression analysis of *SsMYB3* and flavonoid-related regulators in transgenic tobacco flowers. Asterisks indicate a statistically significant difference between wild-type and transgenic plants (*P*≤ 0.05 by Student’s *t*-test).

To test the regulatory function of *SsMYB3* in tobacco, a 35S::*SsMYB3* construct was transferred into tobacco by *Agrobacterium*-mediated transformation of leaf discs, and several transgenic lines were generated in which the flowers showed a visible decrease in color (Fig 5A). Compared with wild-type control plants that produced pink flowers, the flower colors of all T_1_ transgenic lines showed clear phenotypic changes in petal pigmentation patterns, from pink to almost white. For example, some lines (e.g., OX-2) displayed very pale pink flowers with pale red veins, and other lines (e.g., OX-15) produced almost-white flowers with a small, pale-pink region on the edge of petal. Further, anthocyanin and CT contents of transgenic and wild-type flowers were measured, respectively; anthocyanin levels of all transgenic lines were significantly lower than those of the wild-type (Fig 5B), while their CT contents were significantly higher (Fig 5C). In each transgenic line, an inverse relationship between anthocyanin and CT level was detected. For example, transgenic line OX-15 accumulated the highest levels of CTs and the lowest levels of anthocyanin. RT-PCR analyses revealed that line OX-15 exhibited the highest transcription levels of *SsMYB3*, while the lowest expression level of this gene was detected in line OX-2, which had the highest anthocyanin and lowest CT content (Fig 5D).

Our results are similar with those of previous studies that overexpression of PA structural gene *ANR*s resulted in a visible decrease of flower color in tobacco. And the degree of reduction of anthocyanin content and increase of CT content was negatively or positively correlated, respectively, with the expression level of *ANR*s gene [3, 7, 9–12]. These combined findings suggest that *SsMYB3* is a functional gene in tobacco and its ectopic expression can promote biosynthesis of CTs, but reduce accumulation of anthocyanins to change flower color form pink to almost white, in transgenic flowers.

### Ectopic expression of *SsMYB3* affects expression of key genes for anthocyanin and PA biosynthesis in transgenic tobacco

To analyze the effects of ectopic expression of *SsMYB3* on TFs involved in biosynthesis of tobacco flavonoids, RT-PCR analysis were performed. The results showed that the overexpression of *SsMYB3* affected expression of the PA-related genes (Fig 5D). The expression levels of two bHLH-type genes, *NtAn1a* [42]and *NtJAF13-1*, showed slight changes, but the R2R3 MYB-type *NtAn2* gene was downregulated and the WD40-type *NtAn11-1* gene was upregulated in all transgenic lines. The overexpression of *SsMYB3* may have competitively inhibited expression of the endogenous same-type gene *NtAn2*, thereby affecting transcription of other genes of the complex.

**Fig 6:**
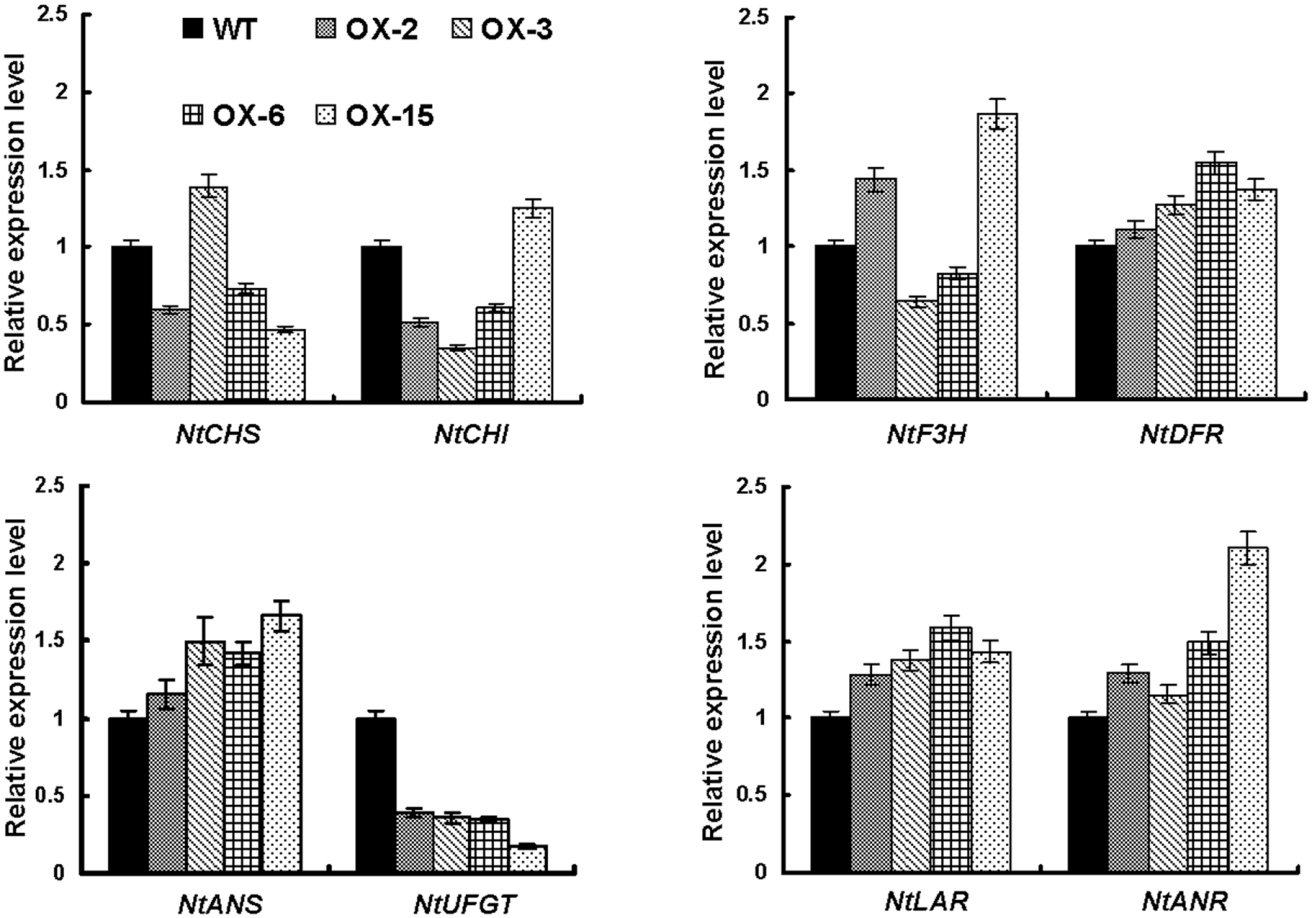
Transcript expression profiles of anthocyanin-related structural genes in flowers of WT and *SsMYB3*-overexpressing transgenic tobacco lines. All transcripts expressed in transgenic flowers were quantified relative to those expressed in WT tobacco flowers (2^-ΔΔCt^).

Further qPCR analysis showed that overexpressing *SsMYB3* strongly influenced expression of the key structural genes of the anthocyanin and PA pathways in transgenic tobacco flowers (Fig 6). For example, expression patterns of early anthocyanin structural genes encoding NtCHS, NtCHI, and NtF3H were complicated and showed no obvious rules in transgenic flowers. These early genes were upregulated in some lines and downregulated in others. Late anthocyanin structural genes (*NtDFR* and *NtANS*) and key PA-biosynthetic genes (*NtLAR* and *NtANR*) were upregulated and showed higher levels of expression in all transgenic lines. However, accumulation of transcripts of *NtUFGT* was significantly lower in all transgenic plants than in the wild type. *NtUFGT* is responsible for the last step inanthocyanin biosynthesis, which transfers the glucosyl moiety from UDP-glucose to the 3-hydroxyl group of anthocyanidins forming stable and water soluble anthocyanins. The loss of function or low expression of *NtUFGT* leads to loss or reduced accumulation of anthocyanin [43–46]. The lowest expression level of *NtUFGT* was found in line OX-15, which produced almost-white flowers with the lowest anthocyanin content. These data indicated that *SsMYB3* can upregulate or activate PA biosynthetic key genes *NtLAR* and *NtANR*, but downregulate or not activate transcript accumulation of the key anthocyanin gene *NtUFGT* in transgenic tobacco flower.

### SsMYB3 may form a temporary transcription complex to regulate PA biosynthesis in transgenic tobacco

**Fig 7:**
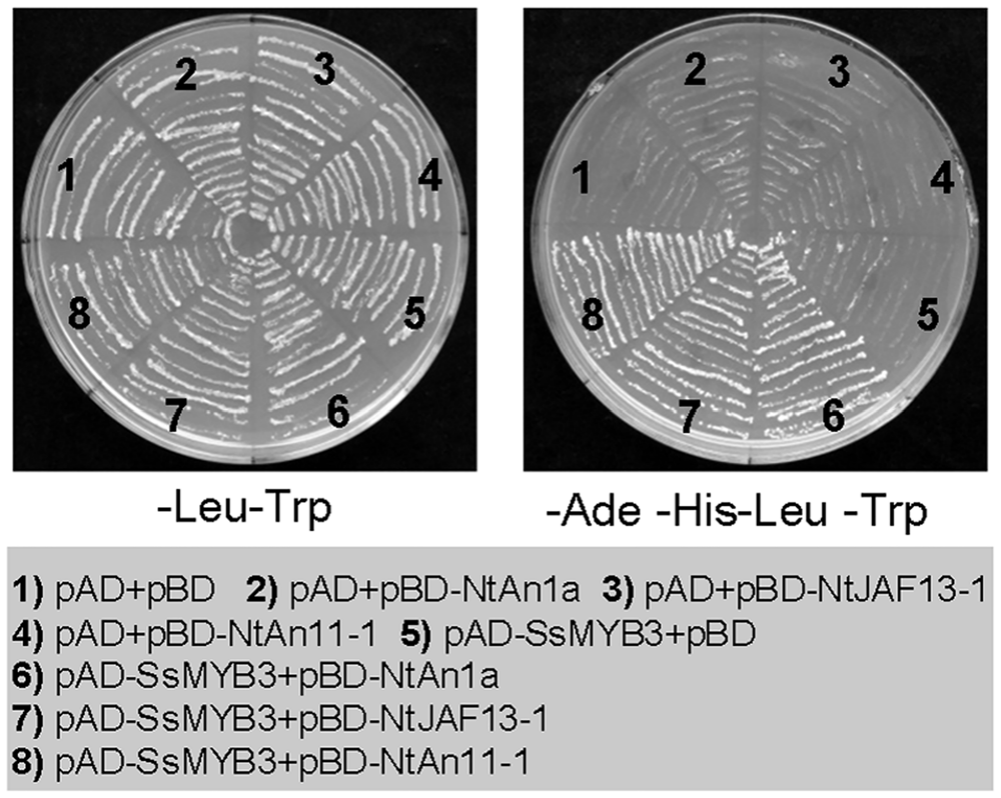
Protein–protein interactions between SsMYB3 and tobacco flavonoid-related regulators. In a yeast two-hybrid assay, the SsMYB3/GAL4-activation domain fusion (pAD-SsMYB3) was co-transformed with fusion constructs of the GAL4-DNA-binding domain with the WD40 protein NtAn11-1 (pBD-NtAn11), the MYB-interaction regions from NtAn1a (pBD-NtAn1a^aa1–195^), or NtJAF13-1 (pBD-NtJAF13-1^aa1-203^).

The tobacco flavonoid regulatory genes *NtAn1*, *NtJAF13*, *NtAn2* [36], and *NtAn11* are homologues of model plant petunia *PhAn1*, *PhJAF13*, *PhAn2*, and *PhAn11*, respectively. Bai et al. [42] and our studies (unpublished) indicated that these regulators can form a protein complex NtAn2-NtAn1**/**NtJAF13-NtAn11 (NtAn2-complex) to control biosynthesis of tobacco flavonoids, like the role of their homologues in petunia [47]. Because the overexpression of *SsMYB3* can rescue the brown phenotype in the PA-deficient *Arabidopsis tt2* mutant seed coat, and SsMYB3 protein can interact with AtTT8 and AtTTG1 in yeast, we speculate that there is a similar interaction between SsMYB3 and tobacco flavonoid regulators. We performed Y2H assays to examine whether SsMYB3 could interact with regulators of flavonoid biosynthesis in tobacco. In yeast, SsMYB3 interacted with two bHLH proteins (NtAn1a and NtJAF13-1) and a WD40 protein NtAn11-1(Fig 7), which implied that SsMYB3 may form a temporary transcription complex SsMYB3-bHLH-WD40 (SsMYB3-complex) to control PA biosynthesis in transgenic tobacco flowers.

**Fig 8:**
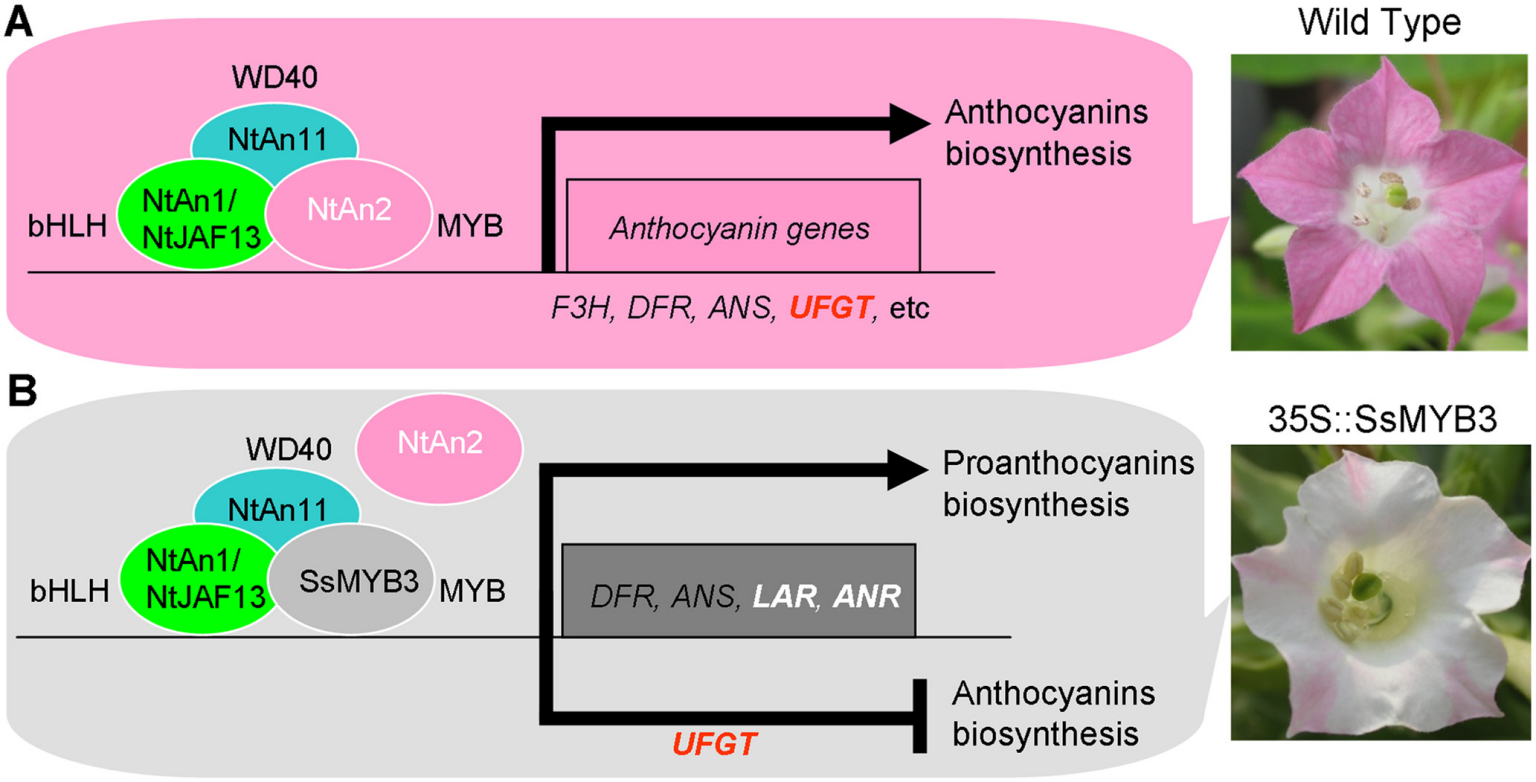
A model for regulation of PAs and anthocyanins biosynthesis by SsMYB3 in transgenic tobacco. In wild tobacco (A), the NtAn2-complex controls anthocyanins biosynthesis by activating expression of late anthocyanin structural genes, such as *F3H*, *DFR*, *ANS*, and *UFGT*. In SsMYB3-overexpressing tobacco (B), high levels of SsMYB3 compete with NtAn2 to form SsMYB3-complex promoting PAs biosynthesis by specially activating expression of *LAR* and *ANR*, and repressing anthocyanins accumulation by inactivating expression of *UFGT*, through inhibition or destabilization of the formation of NtAn2-complex.

On the basis of these analyses, we propose a model for how CTs content, anthocyanins accumulation and flower color are affected by a PA regulator SsMYB3 in transgenic tobacco (Fig 8). The exogenous SsMYB3 may compete with endogenous anthocyanin regulator NtAn2 for binding to the bHLH regulators (*NtAn1* ***/*** *NtJAF13*), and the formative SsMYB3-complex may inhibit or destabilize the formation of NtAn2-complex in *SsMYB3*-overexpressing transgenic tobacco flowers. As a result of this competition, the SsMYB3-complex may specially activate or upregulate expression levels of key PA-biosynthetic genes *NtLAR* and *NtANR* to increase the content of CTs, but the anthocyanin final key gene *NtUFGT* could not be activated. Meanwhile, the lack of NtAn2-complex may result in lower expression levels of the *NtUFGT*. The low levels of *NtUFGT* directly lead to an almost white-flowered phenotype with limited accumulation of anthocyanins. Furthermore, the reduced biosynthesis of anthocyanin probably provided more substrates for biosynthesis of CTs.

This was similar to findings for PA regulators AtTT2, VvMYBPA1 and nectarine/peach PpMYBPA1. In *Arabidopsis*, the MYB-type regulator AtPAP1/2 and AtTT2 can interact with AtTT8 and AtTTG1 to form different transcription complexes that regulate anthocyanin and PA biosynthesis, respectively [13]. The key late anthocyanin structural gene *AtUFGT* can be specially activated by the AtPAP1/2-complex rather than the AtTT2-complex [48]. In grapevine, VvMYBPA1 controls key PA-biosynthetic genes *VvLAR* and *VvANR*, but cannot regulate the key late anthocyanin structural gene *VvUFGT* [17]. In nectarine (*Prunus persica*), PpMYBPA1 trans-activates the promoters of PA pathway genes *DFR* and *LAR*, but not *UFGT* [49].

Over all, overexpression of *SsMYB3* in tobacco results in a significant white flower phenotype with high CT content and low accumulation of anthocyanins. On the basis of functional characterization of *SsMYB3* in the *Arabidopsis tt2* mutant and transgenic tobacco, we concluded that *SsMYB3* gene is an R2R3 MYB-type PA regulator involved in the regulation of PA biosynthesis in coleus. This study contributes to our understanding of regulation of secondary metabolites in coleus, and provides a potential molecular tool for enhancing PA biosynthesis in other fruits and crops using metabolic engineering.

## Supporting Information

S1 Fig Isolation of the *SsMYB3* gene by RACE amplification (A) and full-length PCR amplification (B). (TIF)

S2 Fig Nucleotide and deduced amino acid sequences of *SsMYB3*. The start codon ATG and stop codon TAG are in bold and underlined; the predicted Kozak sequence including ATG is boxed and introns are underlined with dashes. The predicted conserved R2R3 domain is denoted with gray background; the conserved bHLH motif is underlined in gray. The presumed polyadenylation signal AATTAA is wave-underlined.
(TIF)

S3 Fig RT-PCR analyses of early structural genes related to flavonoid biosynthesis in Arabidopsis WT, *tt2* mutant, and transgenic lines. (TIF)

S1 Table List of primers used for *SsMYB3* isolation and characterization. (PDF)

S2 Table List of primers used for the RT-PCR assay. (PDF)

S3 Table List of primers used for the qPCR assay. (PDF)
