# Supplementary figures and images for: Ectopic Expression of the Coleus R2R3 MYB-Type Proanthocyanidin Regulator Gene SsMYB3 Alters the Flower Color in Transgenic Tobacco
Source: PLoS One. 2015 Oct 8;10(10):e0139392. doi: 10.1371/journal.pone.0139392 (PMC4598174; doi:10.1371/journal.pone.0139392)

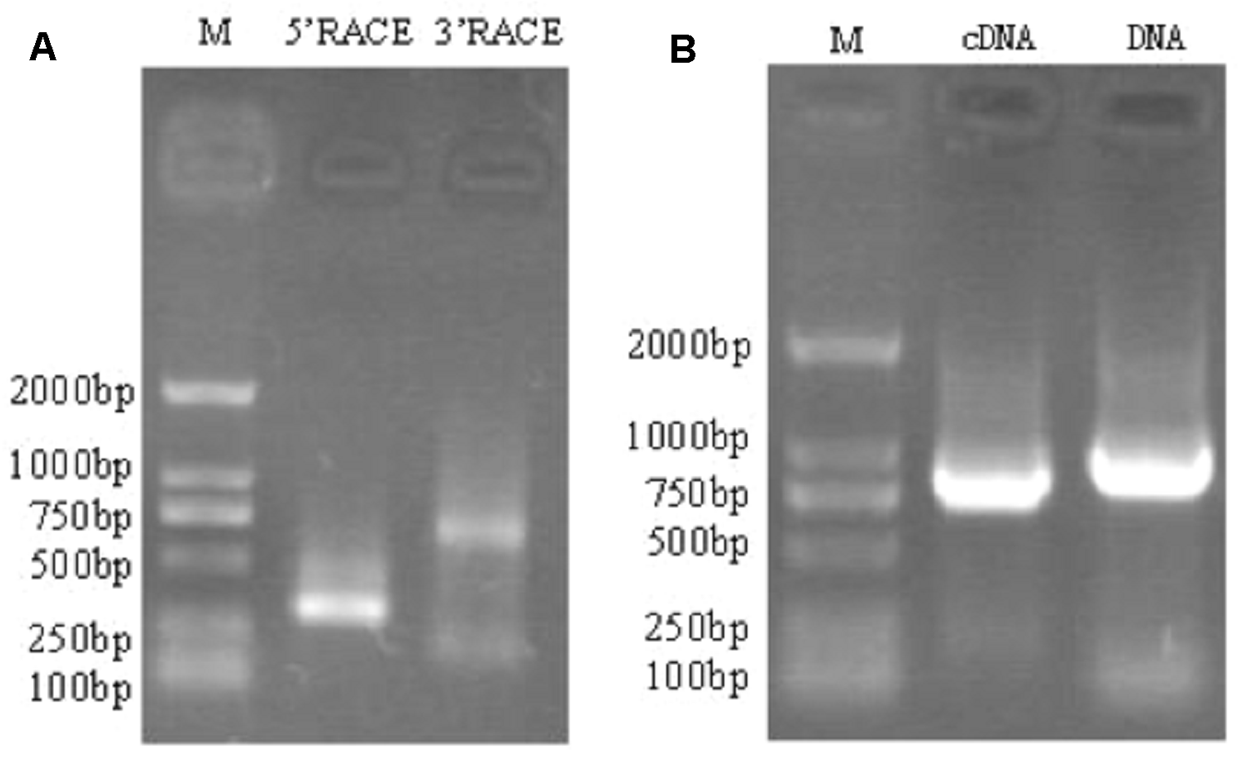

Supplement: S1 Fig — (TIF) [file pone.0139392.s001.TIF]

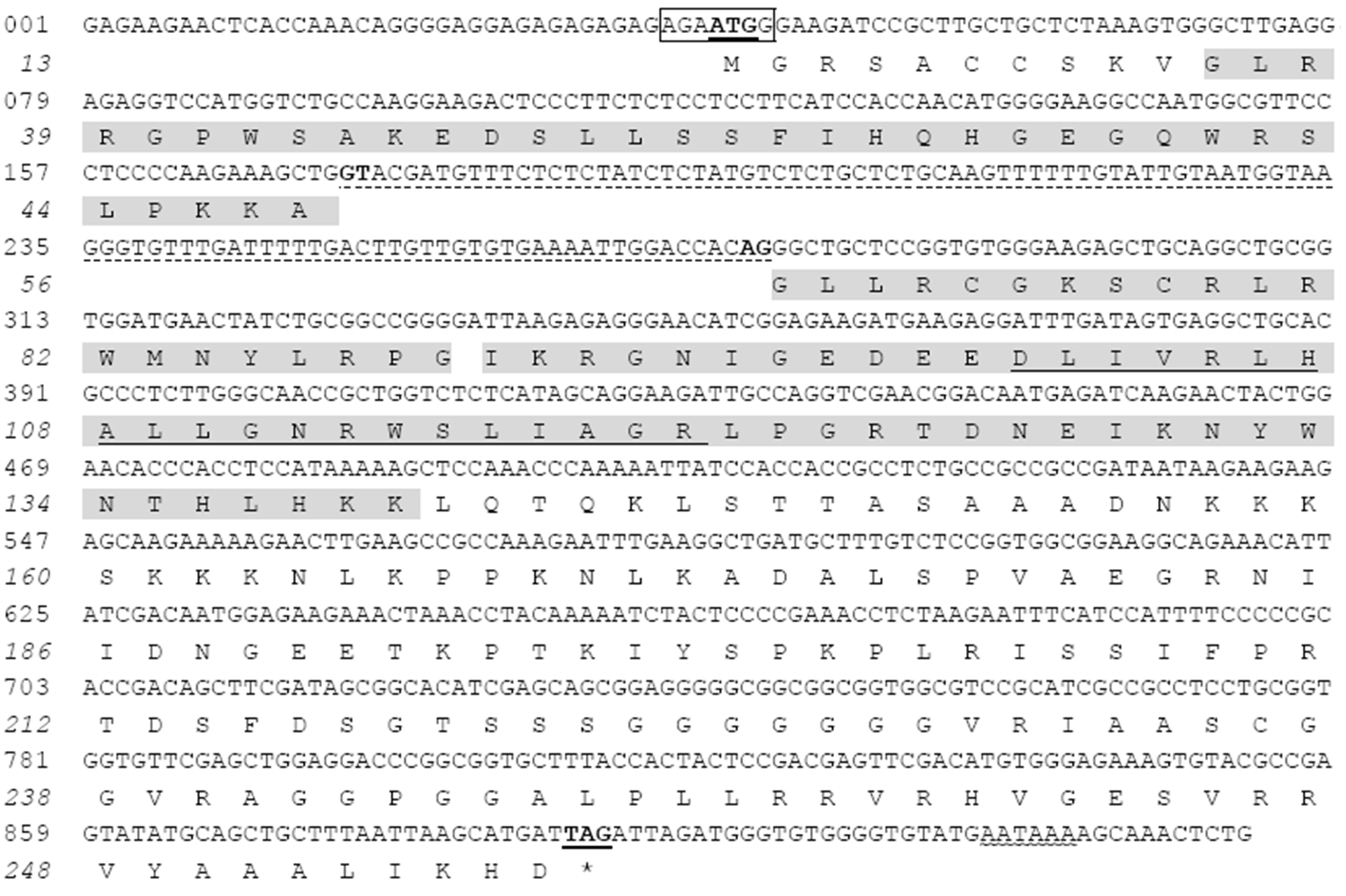

Supplement: S2 Fig — The start codon ATG and stop codon TAG are in bold and underlined; the predicted Kozak sequence including ATG is boxed and introns are underlined with dashes. The predicted conserved R2R3 domain is denoted with gray background; the conserved bHLH motif is underlined in gray. The presumed polyadenylation signal AATTAA is wave-underlined. (TIF) [file pone.0139392.s002.TIF]

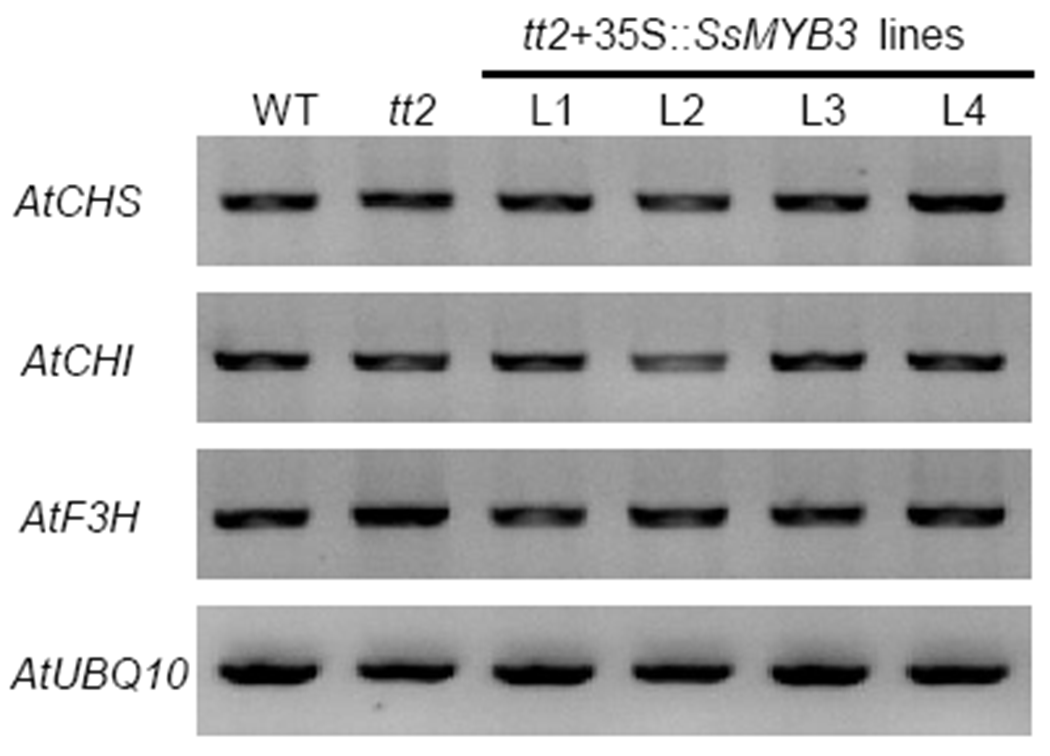

Supplement: S3 Fig — (TIF) [file pone.0139392.s003.TIF]
