# Supplementary material for: Ectopic Expression of the Coleus R2R3 MYB-Type Proanthocyanidin Regulator Gene SsMYB3 Alters the Flower Color in Transgenic Tobacco
Source: PLoS One. 2015 Oct 8;10(10):e0139392. doi: 10.1371/journal.pone.0139392 (PMC4598174; doi:10.1371/journal.pone.0139392)
Supplement: S1 Table — (PDF) [file pone.0139392.s004.pdf]

S1 Table List of primers used for Ss*MYB3* isolation and characterization.

**Name**	**Primer (5'- 3')**	**Note**
FdMYB	TGYRGVAARAGYTGYMGVCWNMGVTGG	Degenerate primers for
RdMYB	GTTCCAVYARTTYTTSAYVKCRTTDKC	*MYB* cDNA
GeneRacer5'P	CGACTGGAGCACGAGGACACTGA	1st round of 5'-RACE
RMYB3-5GSP1	GACCTGGCAATCTTCCTGCTATGAG	PCR
GeneRacer 5'NP	GGACACTGACATGGACTGAAGGAGTA	2nd round of 5'-RACE
RMYB3-5GSP2	CGTGCAGCCTCACTATCAAATCCTC	PCR
GeneRacer 3'P	GCTGTCAACGATACGCTACGTAACG	1st round of 3'-RACE
FMYB3-3GSP1	GGGA TTAAGAGAGGGAACATCGGAG	PCR
GeneRacer 3'NP	CGCTACGTAACGGCATGACAGTG	2nd round of 3'-RACE
FMYB3-3GSP2	CCTCTTGGGCAACCGCTGGTCT CTC	PCR
FMYB3	ATAATTCTAGAGAAGAACTCACCAAACAGGG [a]	Full length PCR
RMYB3	AATAACCCGGGCAGAGTTTGCTTTTATTCATACACC [a]	

[a] indicates the additional restriction enzyme site are underlined.
