# Supplementary material for: Ectopic Expression of the Coleus R2R3 MYB-Type Proanthocyanidin Regulator Gene SsMYB3 Alters the Flower Color in Transgenic Tobacco
Source: PLoS One. 2015 Oct 8;10(10):e0139392. doi: 10.1371/journal.pone.0139392 (PMC4598174; doi:10.1371/journal.pone.0139392)
Supplement: S2 Table — (PDF) [file pone.0139392.s005.pdf]

S2 Table List of primers used for RT-PCR assay.

Gene name	Accession number	Forward primer (5'-3')	Reverse primer (5'-3')
SsMYB3	EF522163	CTACTGGAACACCCACCTC	CGAACTCGTCGGAGTAGTG
AtCHS	AT5G13930	CCATCTCCTCAAGGATGTTC	CTGACTTCCTCCTCATCTCG
AtCHI	AT3G55120	TGACAATGAAACTGCCGTTAAC	CTAGTGCCAGGTGACACACC
AtF3H	AT3G51240	CAATGCATGCGTCGATATGG	GTGGTCGCCGAGATTGACGAC
AtDFR	AT5G42800	CTCTCCTATCACTCGGAACGAG	AGTTAAACCCCATGTCCGTCAG
AtANS	AT4G22880	TACGAGGGCAAATGGGTCAC	GAGCAAAAGTCCGTGGAGGA
AtANR	AT1G61720	TGCAGAAGCTATCTGGCTCG	TTCGGAATCGACAAGCCCTC
AtUBQ10	AT4G05320	AGAGCGTCTCATCTTCGCTG	GCCCCAAAACACAAACCACC
NtAn1a	HQ589208	TCGCCACTGTTGATTCCTCC	CCTTCACTGTAGCTGTGCCA
NtJAF13a	FG622526	CGCCAAGAATTGGAACTGCAC	TTTGTCAACCTTGCCACCAG
NtAn2	FJ472647	AAGGACGGCAAACGATGTCA	AGCTAGTAAATCGGCCCACC
NtAn11-1	FJ795022	ATGCGATTGCGTGGGCTCCACA	CAAGCAATCCAAAAGGGTTGCA
NtGAPDH	AJ133422	G GATTAGTGGCCAGGGTTGCT	C ACCACCCTTCAAGTGAGCAG
