# Supplementary material for: Ectopic Expression of the Coleus R2R3 MYB-Type Proanthocyanidin Regulator Gene SsMYB3 Alters the Flower Color in Transgenic Tobacco
Source: PLoS One. 2015 Oct 8;10(10):e0139392. doi: 10.1371/journal.pone.0139392 (PMC4598174; doi:10.1371/journal.pone.0139392)
Supplement: S3 Table — (PDF) [file pone.0139392.s006.pdf]

S3 Table List of primers used for qPCR assay

Gene name	Accession number	Forward primer (5'-3')	Reverse primer (5'-3')
SsMYB3	EF522163	TGGCGGAAGGCAGAAACATT	TGCGGGGGAAAATGGATGAA
SsACT	DQ423374	GGCTTACACCATCACCAGAGT	CCAAGGCGAACAGAGAGAAA
NtCHS	AF311783	ACTCCGGATGGCTAAGGACT	ACCTATAATGACCGCGGCTG
NtCHI	AB213651	ACTGGCACTGGAAATGCTGT	AAACTGACGCGTCGGCATAG
NtF3H	AB289450	AAGGCAGTAATGGACGAGC	TGGCCTTCTCAGCAGCTTTT
NtDFR	EF421429	GGGAATGAAGCTCACTACAGC	ACTCCGGCCATTTCTCTTGG
NtANS	AB289447	TGTCCCCAACCAGAACTAGC	TTTGCCGTTACCCACTGTCC
NtUFGT	FG627024	TTTCGGGGACCAAAAGCTGA	CTGAAAAAGGCATCCAATGCAC
NtLAR	AM827419	TCAATGGTGCGAAAGGACTC	TGCTGCAGAGAATATCAACC
NtANR	AM791704	TGCGGCCGCTGTAAAATTG	CGCGACAAACATCTTCCAC
NtGAPDH	AJ42133422	CTGCTCACTTGAAGGGTGGT	GGGAGCAAGGCAATTTGTGG
